# Classification of Bee Pollen and Prediction of Sensory and Colorimetric Attributes—A Sensometric Fusion Approach by e-Nose, e-Tongue and NIR

**DOI:** 10.3390/s20236768

**Published:** 2020-11-26

**Authors:** László Sipos, Rita Végh, Zsanett Bodor, John-Lewis Zinia Zaukuu, Géza Hitka, György Bázár, Zoltan Kovacs

**Affiliations:** 1 Commercial and Sensory Science, Department of Postharvest, Faculty of Food Science, Szent István University, 39–43 Villányi Street, 1118 Budapest, Hungary; Hitka.Geza@etk.szie.hu; 2 Department of Food Chemistry and Nutrition Science, Faculty of Food Science, Szent István University, 14–16 Somlói Street, 1118 Budapest, Hungary; vegh.rita@phd.uni-szie.hu; 3 Department of Measurement and Process Control, Faculty of Food Science, Szent István University, 14–16 Somlói Street, 1118 Budapest, Hungary; Bodor.Zsanett@hallgato.uni-szie.hu (Z.B.); zaukuu.john-lewis.zinia@hallgato.uni-szie.hu (J.-L.Z.Z.); Kovacs.Zoltan.food@szie.hu (Z.K.); 4 Department of Nutritional Science and Production Technology, Faculty of Agricultural and Environmental Sciences, Szent István University, 40 Guba Sándor Street, 7400 Kaposvár, Hungary; bazar@agrilab.hu; 5 ADEXGO Ltd., 13 Lapostelki Street, 8230 Balatonfüred, Hungary

**Keywords:** CIE L*a*b* colour coordinates, spectra, palynological analysis, electronic nose, electronic tongue, sensory panel performance, multivariate analysis, principal component analysis (PCA), linear discriminant analysis (LDA), partial least square regression (PLSR)

## Abstract

The chemical composition of bee pollens differs greatly and depends primarily on the botanical origin of the product. Therefore, it is a crucially important task to discriminate pollens of different plant species. In our work, we aim to determine the applicability of microscopic pollen analysis, spectral colour measurement, sensory, NIR spectroscopy, e-nose and e-tongue methods for the classification of bee pollen of five different botanical origins. Chemometric methods (PCA, LDA) were used to classify bee pollen loads by analysing the statistical pattern of the samples and to determine the independent and combined effects of the above-mentioned methods. The results of the microscopic analysis identified 100% of sunflower, red clover, rapeseed and two polyfloral pollens mainly containing lakeshore bulrush and spiny plumeless thistle. The colour profiles of the samples were different for the five different samples. E-nose and NIR provided 100% classification accuracy, while e-tongue > 94% classification accuracy for the botanical origin identification using LDA. Partial least square regression (PLS) results built to regress on the sensory and spectral colour attributes using the fused data of NIR spectroscopy, e-nose and e-tongue showed higher than 0.8 R^2^ during the validation except for one attribute, which was much higher compared to the independent models built for instruments.

## 1. Introduction

**Figure 1:**
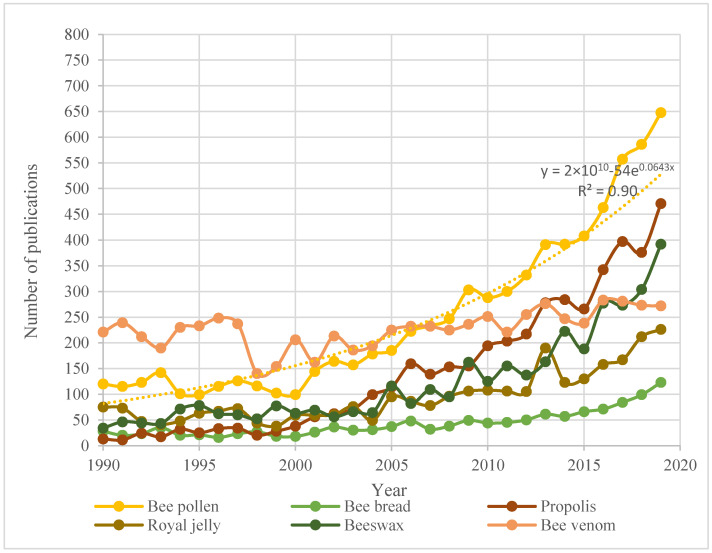
Temporal trend of scientific articles published on the topic of apicultural products except for honey [2].

The increasing prominence of alternative and complementary medicine in the 21st century has emphasized the growing interest in the protection of insect pollinators. Consequently, a widening range of apicultural products is available in the market. According to Bargańska et al. [1], these products can be classified into two groups according to their origin: honey, bee pollen, bee bread, and propolis are of vegetable origin, while royal jelly, beeswax, and bee venom are secretions of bees. The market growth of beekeeping products entailed a large increase in the number of scientific articles relating to this topic. Figure 1 shows the temporal trend of scientific articles published in the above-mentioned domain between the years of 1990 and 2019, based on ScienceDirect search results [2]. The number of published papers in the last two years is approximately on par with those published in the 1990s, and the number of scientific studies on bee pollen has grown exponentially over the last 30 years.

Besides honey, bee pollen is a popular beekeeping product which can be produced at a relatively low price and can be consumed in many forms. According to Kieliszek et al. [3], the global production of bee pollen is around 1500 tons. According to Ulbricht et al [4], bee pollen is consumed by followers of a health-conscious lifestyle as it contains nutrients and various bioactive compounds in a relatively large concentration and also in a balanced proportion. Thakur and Nada [5] determined the average composition of pollen loads based on above 100 studies. Data of both fresh and dried products were included in the studies. According to their results, bee pollen on average contains 54.22% (18.50–84.25%) carbohydrates, 21.30% (4.50–40.70%) proteins, 5.31% (0.41–13.50%) lipids, 8.75% (0.15–31.26%) fibre and 2.91% (0.50–7.75%) ash. These products are valuable sources of essential micronutrients like minerals [6,7,8], vitamins [9,10], and phenolic compounds [6,9]. Major contaminants of the pollen products are pesticides [11,12], toxic metals [13,14], moulds and mycotoxins [15]. Other possible contaminants of the product are polychlorinated biphenyls [16,17], polybrominated diphenyl ethers [17], polycyclic aromatic hydrocarbons [18], nanosized iron-oxides, iron-hydroxides, and barytes [19].

In 2019, the Technical Committee for Food Products of the International Organization for Standardization established a subcommittee on beekeeping products (ISO/TC34/SC19). Within the subcommittee, working groups have been formed for honey (ISO/TC34/SC19/WG1), propolis (ISO/TC34/SC19/WG2), royal jelly (ISO/TC34/SC19/WG4) and bee pollen (ISO/TC34/SC19/WG3). The latter group recently began standardizing bee pollen, and the document currently under development suggests the following tests for the product: moisture, total protein, total sugar, total lipid and ash content.

The pollen foraging behaviour of bees is influenced by the following factors: abundance and diversity of floral resources [20], availability of pollen grains, season [21], time of the day, temperature, number of larvae present in the hive, amount of stored food, nutrient (especially protein) quality and quantity of the pollen [22], colour, shape, morphology and odour of the flower [23], health status of the bees [24], and nectar quality [25]. As bees collect pollen from a single plant species at a given time, colours of pollen loads are homogenous. Different types of pollen loads show a wide range of colours, but they are shades of yellow and orange in most cases [26].

Environmental conditions are generally not suitable for harvesting monofloral bee pollen. Beekeepers, therefore, collect polyfloral pollen in most cases, which is difficult to characterize. Pollen loads from different plant species differ largely in their nutritional, sensory, and food safety attributes. Thus, the identification and characterization of pollen loads are crucially important for food development and public health.

Microscopic pollen analysis is commonly used to determine the botanical origin of pollen. Pollen analysis of honey is also a useful tool in the identification of botanical origin, and in some cases, the geographical origin. This is because honey contains pollen from the plants as a result of bees collecting nectar. Generally, the botanical origin of honey is determined according to the concentration of the main pollen in the sample; however, for the exact determination of the origin, the sensory and physicochemical properties are also needed [27,28]. Identification of pollen grains is currently performed by the visual inspection of microscopy images. It requires a trained expert to manually classify each pollen grain based on its size, shape, texture and other morphological properties. Consequently, this method is very time-consuming and costly. Furthermore, since the obtained result depends on the expert’s criterion, this technique is less objective than automated methods [29]. Accurate identification is aided by standards and microscopic pollen databases, e.g., the Palynological Pollen Database [30], Global Pollen Database [31], European Pollen Database [32], the Pollen and spores image database of Department of Palynology and Climate Dynamics, Georg-August-Univesität [33].

In the practice of food sciences, many tools and techniques can be used to distinguish different samples: for example, sensory tests, near-infrared (NIR) spectroscopy, spectral colour measurement, and electronic sensors (electronic tongue, electronic nose).

Sensory profiling is a description of the sensory properties of a sample, usually based on the evaluation of sensory properties by assigning an intensity value to each property. Quantitative profile analysis is one of the most commonly used techniques that can be used to fully characterize products. The advantage of sensory profiling is that it can be easily linked to other instrumental, chemical, or physical properties using multivariate statistical methods. In this way, the instrumental values of the evaluated sensory property can be determined, and the mapping of the products can become complete [34,35,36,37].

Near-infrared (NIR) spectroscopy is a well-established technique that uses the near-infrared range (780–2500 nm) of the electromagnetic spectrum. In NIR spectroscopy, the sample is irradiated with near-infrared radiation, and the reflected or transmitted radiation is measured. As a result of the interaction of near-infrared electromagnetic waves with the sample constituents, the spectral characteristics of the sample change through scattering and absorption processes. This change is composition-dependent [38]; consequently, this technique is suitable for both qualitative and quantitative analysis. Major advantages of NIR spectroscopy are that it requires little to no preparation, its rapidness, safety to the analyst, non-destructiveness, and multicomponent remote analysis [39]. Most molecules in foods contain C-H, N-H, S-H, or O-H bonds, so the application of NIR spectroscopy is nearly universal. On the other hand, this technology is heavily dependent on reference conventional methods to develop a calibration model and validate it [40].

According to Pathare et al [41], food colour is the first sensation that consumers perceive and it greatly influences their decision to purchase. Colour perception depends on the observer and the conditions in which the colour is observed. Consequently, the objective characterization of the colour of different products and quantification of the differences between them is an important area of research. The colour of an object can be described by colour coordinate systems. The Commission Internationale de l’Éclairages (CIE) system is based on the concept that the human eye has three colour receptors (red, green and blue) and all colours are combinations of those. CIELAB colour scales are commonly used in the food industry. The CIELAB colour coordinates are a* (takes positive values for reddish colours and negative values for the greenish ones) and b* (takes positive values for yellowish colours and negative values for the bluish ones). L* express lightness on the grey-scale, from absolute black to absolute white. The chroma (C*) is considered the quantitative attribute of colourfulness (intensity) of samples perceived by humans. The hue angle (h*) is the attribute which defines the difference of a certain colour with reference to grey colour with the same lightness. The CIELAB system provides the ability to define the colour of objects easily, effectively, and reliably [42].

The electronic nose (e-nose) and electronic tongue (e-tongue) are promising tools in the field of food authenticity assessment. Both are sensor systems, but they work differently: the former is invasive, while the latter is non-invasive. E-tongues are groups of sensors with partial specificity and a pattern recognition system. The response of all the sensors can be interpreted as the fingerprint of a particular sample which can be used to identify and classify the tastes of liquid phase samples. Electronic noses generally consist of an array of sensors utilized to detect and distinguish odours of samples. Electronic nose (e-nose) systems for foodstuffs are very attractive due to the simplicity of analysis, the minimal sample preparation required, and the proximity of the approach in odour assessments to the sensory testing [43]. These instruments enable cost-effective and rapid measurements of complex samples that can be utilized for both qualitative and quantitative purposes [44,45]. The main limitation of these systems is that they are strongly affected by environmental conditions. The E-tongue is influenced by temperature, while the e-nose is affected by both temperature and humidity. The advantages of e-tongues over e-noses are higher selectivity and significantly lower detection limits [46].

In recent years, more and more international research has focused on integral examination of products. We can get more information of the samples by applying several methods simultaneously than by applying a single method. Due to the high complexity of food, the employment of just single sensor data is often insufficient, and multisensor data fusion techniques, combining the outputs of multiple instrumental sources, have been developed [47]. The efficiency of the fusion signals outperformed that of independent signals [48]. Multivariate chemometric methods such as the principal component analysis (PCA), linear discriminant analysis (LDA), partial least square regression (PLSR), and artificial neural networks (ANN) are among those typically used for this purpose [49]. To date, only a few studies have been published on the topic of bee pollen classification by integrated methods. Salazar-González et al. [50] developed a methodology to classify pollen loads of different origins based on their harvest month, colorimetric properties, and particle size. Thakur and Nada [51] performed a PCA using four monofloral and one polyfloral bee pollen samples to assure that the physical, functional, and textural properties could be used to visualize differential patterns of samples as per their botanical origin. Castiglioni et al [26] examined the relationships between morphological, spectroscopic, and colour properties of pollen loads belonging to different botanical origins. They used multivariate analysis to differentiate and classify the samples according to their origin.

In our work, we aim to determine the applicability of microscopic pollen analysis, spectral colour measurement, NIR spectroscopy, e-nose, e-tongue, and sensory methods for the classification of bee pollen of different botanical origin. A further goal was to build chemometric models for the prediction of sensory and colour parameters of pollen independently with NIR spectroscopy, the e-nose, e-tongue, and their fusion matrix.

## 2. Materials and Methods

### 2.1. Materials

In our study, dried bee pollen samples of five different botanical origins from the apiary of Bertalan Imre, Püski were examined. The samples were purchased in a Hungarian retail store in 2020 and stored in a dark place at room temperature (20 °C ± 2 °C) until use.

### 2.2. Microscopic Pollen Analysis

Pollen loads of the samples were sorted by colour, shape, and size. As a result, five samples with different shades (dark orange, bright orange, brown, matte yellow, dark reddish-brown) were obtained, and their botanical compositions were determined via microscopic pollen analysis. The determination was performed by a member of the Melissopalynological Group of the International Honey Commission (IHC) with the following method: 10 pollen loads of each sample were suspended in 10 mL water in a 15 mL centrifuge tube. Pollen grains were dispersed completely by using a test tube mixer. The suspension (30 µL) was transferred onto two slides using a micropipette. After drying on a hot plate, they were covered with glycerine gelatine mixture and glycerine gelatine mixture stained with fuchsine. Pollen grain identification was performed for both slides by examining the entire area of a 20 × 20 mm cover slip. A Delta Optical binocular light microscope (Delta Optical, Warsaw, Poland) at 400× magnifications was used for the determination.

### 2.3. Spectral Colour Measurement (L*a*b* System)

The colour measurement was performed with a Konica Minolta chroma meter CR-400 colour measuring device equipped with six highly sensitive silicon photodiodes. During the measurement, the sample was illuminated by a xenon lamp on an 8 mm diameter circular surface. Prior to the assay, the device was calibrated using a white standard (L* = 87.2; a* = 3.11; b* = 3.17). After grinding and homogenization, approximately 3 g of each sample was placed in the sample holder in a layer 1 cm thick. Thus, the sample completely covered the surface corresponding to the size of the measuring apparatus. The measuring apparatus was lowered to the bottom of the sample holder before the measurement was started. The L*, a* and b* values were obtained after a few seconds of the sample measurement. Ten parallel measurements were performed for each sample (5 samples × 10 replicates = 50 recording per colour values). Derived from the tristimulus values L*, a*, b* coordinates and ΔE*_ab_ colour differences, chroma and hue angle were calculated according to Commission Internationale de l’Éclairage [52].

### 2.4. Sensory Analysis

Our tests were based on standard sensory methods and performance indicators, international software used in sensory practices, and the tests were performed similarly to our previous research [53]. Sensory evaluations were conducted at Szent István University, Sensory Evaluation Laboratory, which meets standard requirements [54]. The panel consisted of 14 trained assessors (7 females and 7 males, between the ages of 20 to 28) with the necessary knowledge and experience in sensory descriptive analysis. This includes techniques and practices in attribute identification and terminology development. These individuals went through training which met the standard requirements [55]. The trained panel sensory tests were carried out using quantitative descriptive profile (QDP) method [37]. The trained panel evaluated the pollens using a scale between 0 and 100 for each, where 0 was the lowest score and 100 was the highest. The panellists analysed 18 attributes, which involved appearance (brightness, colour hue, homogeneity of the surface), odour (global odour intensity, sweet odour intensity, sour odour intensity, floral odour intensity, hay odour intensity), taste/flavour (global taste intensity, sweet taste intensity, sour taste intensity, floral taste intensity, hay flavour intensity, off-taste intensity, after taste intensity), and texture (hardness, cohesiveness, mouthcoating). A separate text box was available to describe other odours and flavours. In order to prevent sensory fatigue, there was a half-hour break between appearance/odour attributes and taste/texture attributes. Five pollen samples were evaluated by the panel. Tests were conducted using two replicates to ensure data reliability. The sessions were conducted on 2 different days to achieve proper repetitions (5 samples × 14 panellists × 2 session = 140 observation per sensory attributes). The sessions were held in the morning (between 10 a.m. and 12 a.m.) because the human senses are the most sensitive during this period [56]. Sensory panellists were proficient in examining apicultural products.

### 2.5. Near Infrared Spectroscopy Measurements (NIR)

Samples were milled in a mortar to facilitate solubility in distilled water. From each sample, a 20-times dilution was prepared. The milled pollen (6 g) was weighted in and filled up to volume in 100 mL volumetric flask, then transferred to beakers, and 20 mL of distilled water was added. The solutions were filtered using filter paper (Macherey-Nagel, 24 cm, Düren, Germany). Each sample was prepared in three repeats. For each sample, 300 μL of the filtrate was used for NIR measurement.

Near-infrared spectroscopy (NIR) measurements were performed using the DLP NIRScanNano instrument (Texas Instruments, Dallas, TX, USA). The transmission spectra of the samples were recorded at the 900–1700 nm spectral range at about a 3 nm spectral step (10 nm optical resolution) with a 1 mm path length in the cuvette. The absorbance spectra were calculated against the factory built-in white ceramic reference standard. Spectra of distilled water were recorded between each of the five samples to monitor the stability of the system during the experiment. Five consecutive scans were recorded for each sample (5 samples × 3 replicates × 5 consecutive scans, *n* = 75), resulting in 95 spectra in total (including distilled water). Results of the distilled water spectra proved the stability of the spectrometer during the experiment; thus, it was not included in the further data analysis.

### 2.6. Electronic Nose Measurements

The Heracles Neo 300 ultra-fast GC analyser (Alpha MOS, Toulouse, France) was used to perform the electronic nose measurement. The Heracles e-nose is designed to analyse the volatile compounds, and it is composed of a selective, rapid, and highly sensitive gas chromatography system. The e-nose system has an odour concentrator that is called the trap. Its operation is completed if the samples are injected and concentrated in the cold trap, after the trap is heated, and the concentrated odour is injected and distributed to the two columns (Restek MXT-5: length 10 m; ID 0.18 mm; thickness; 0.40 μm and Restek MXT-1701: length 10 m ID 0.18 mm; thickness; 0.40 μm (Restek, Co., Bellefonte, PA, USA). The columns are metal capillary columns; MXT-5 is composed of Crossbond 5% diphenyl/95% dimethyl polysiloxane), while MXT-1701 composed of Crossbond 14%cyanopropylphenyl/86% dimethyl polysiloxane. The volatile compounds separated by the columns are detected using two flame ionization detectors (one for each column). AlphaSoft v16 (Alpha MOS, Toulouse, France) software was used to operate the autosampler and analyzer, including the data acquisition and basis data transformations. The retention time of the volatiles was recorded throughout the data acquisition, where retention time is characterized by the elution time of the molecules. Retention indices were obtained through conversion from the retention time. Kovats retention index compares the retention time of the investigated volatile molecules of a sample with the retention time of n-alkanes under the same conditions [57]. The Kovats index (KI) characterizes the volatile compounds on the specific columns and can be connected to specific aromas that are collected in the AroChemBase v7 (Toulouse, France) [58]. Throughout the manuscript, as an identifier, the “1A” appears for column MXT 5 and “2A” for column MXT 1701 after the KI.

Samples prepared for the NIR spectroscopy analysis were used in the electronic nose measurement. Measurements were performed four different ways to check which method was the most suitable for further analysis and evaluation. 

For the method 1, 3 mL of the sample was heated at 40 °C, for method 2, 5 mL of the sample was heated at 40 °C, for method 3, 3 mL of the sample heated at 60 °C, and for method 4, 5 mL of the sample was heated at 60 °C. Each sample was analysed once, resulting in three replicate measurements for the five botanical groups (5 samples × 3 replicates *n* = 15 observation per method, × 4 methods in total 60 observations).

### 2.7. Electronic Tongue Measurements

Electronic tongue (e-tongue) measurements were performed with an αAstree electronic tongue (AlphaMOS, Toulouse, France), designed for the determination of taste patterns of liquid food samples [59]. The sensor set consists of an Ag/AgCl reference electrode, and seven ISFET (ion-sensitive field-effect transistor) sensors (ZZ, JE, JB, HA, HA, CA, BB), developed for food applications.

For the measurements, 100 mL of each sample filtrate was used and the electrodes were conditioned according to the suggestion of the developers before commencing the experiment. This step was followed by the calibration. The calibration solution was mixed from the prepared samples, containing equal volume from each of the 15 prepared samples. The potential difference between the reference electrode and the seven individual electrodes was measured through 120 s of sample measurement until the sensor signals reach the stable saturation. The average of the last 10 s was calculated and saved as an observation for each sensor per sample. Each sample was measured four times, resulting in 12 observations per the main five samples (5 samples × 3 replicates *n* = 15 × 4 sensor signal obtaining = total 60 observation).

### 2.8. Statistical Analysis

For the evaluation of spectral colour measurement, the mean tristimulus values L*, a*, b* coordinates of bee pollen were compared using one-way analysis of variance (α = 0.05), and the pair comparison was done using Tukey HSD post-hoc test. These tests were carried out using XL-STAT software created by Addinsoft (Paris, France).

The sensory attributes of the pollens were evaluated separately. The mean values were compared by 2-way analysis of variance when evaluating the sensory attributes of the products (α = 0.05). The pair comparison was done via Tukey HSD post-hoc test. These tests were carried out using XL-STAT software created by Addinsoft. The performance monitoring of the panel was carried out according to the workflow of the PanelCheck v1.4.2 software (Ålesund, Norway) using one-and multi-way statistical methods [60,61]. The performance of the trained sensory panel was analysed by mixed assessor model–control of assessor performance (MAM-CAP) table method for testing discrimination, agreement, repeatability, and scaling at the panel. The MAM-CAP table was created in the R-project software v4.0.2 (Vienna, Austria) with MAM-CAP-package [62,63].

For the NIR spectroscopy data set, Savitzky-Golay smoothing (2nd order polynomial, 21 filter length, no derivation) was used to decrease the noise of the acquired spectra, and multiplicative scatter correction (MSC) was applied to avoid baseline shift. The truncated spectral range between the 950–1650 nm interval was used for the NIR data processing to avoid the noisy ranges at the ends of the spectrum. For the e-tongue, a drift correction method called “additive correction relative to all samples” was applied on the data set to reduce the effect of ageing on sensor signals. The method was developed by Kovacs et al [64].

For the e-nose, the 20 most discriminating sensors using the Alphasoft v16 (Toulouse, France) were chosen for further analysis. This was done on the basis of the sensor discrimination powers given by the Alphasoft v16 software. Discrimination power shows the minimum variability within the group and the maximum variability between groups. The results of the chosen sensors were analysed for all the four methods and also separately for each method to be able to find the most useful method in pollen discrimination.

The fusion of the data was also created by pairing the sample results of NIR spectroscopy, e-tongue, and e-nose. In the case of the drift corrected e-tongue data, the respective samples were paired with the pretreated NIR spectra such that the spectra and sensor signal were matched in the same time-order. As NIR contained more observations per sample in the case of e-nose, the average of the sensor values was used as needed, while in the case of E-nose, the results obtained using method 4 were inserted as many times as needed (method 4 was chosen because it had the best discrimination power). Lastly, mean-centering and scaling of the data were applied before model developments. Mean-centering was performed by subtracting the mean value of each variable (wavelengths or sensors) from the respected single values. Then, for scaling, the mean-centered values were divided by the standard deviation of the corresponding variable.

For all the three analytical instruments (NIR spectroscopy, e-tongue and e-nose), multivariate statistical tools were used to build models. Principal component analysis was used to determine patterns in the dataset and to detect outliers by visual inspection. In the case of NIR spectroscopy and e-tongue, ten and five outliers were removed, resulting in 65 and 55 observations, respectively. Linear discriminant analysis (LDA) using threefold cross-validation was used to build classification models according to the botanical origin (noted as validation in the figures). Partial least square regression (PLSR) was applied to regress on the sensory and colour parameters using leave one sample out cross-validation (in this case all observation of one sample was left out and used as a validation data set, while the rest was used to train the model. The cross-validation process was repeated *n* times, where *n* was the number of the samples, in our case *n* = 15 for the 3 replicates of the 5 five samples). The determination coefficient used is noted as R^2^tr and R^2^Cv for training and cross-validation, respectively. Root mean square error (RMSEC) in the case of training and RMSECV in the case of validation was applied to calculate the accuracy and error of the regression. Residual prediction deviation (RPD) was calculated to check the robustness of the model. RPD can be defined as the standard deviation of observed values divided by the root mean square error of prediction (RMSECV). The higher the RPD, the better the model’s predictive ability. PLSR models were also built for the fused data to regress on the sensory attributes and colour parameters [65]. The PLSR models were built in two rounds. The result of the prediction of the first round of PLSR analysis was tested for further outliers. In the PLSR prediction-based outlier detection, boxplot analysis was performed at each level of the predicted parameters, and the observations which lie beyond the extremes of the whiskers defined by 1.5 times the interquartile range from the median were identified as outliers. These outliers were omitted before the second round of PLSR modelling. Our PLRS code contains an outlier detection method based on a boxplot analysis of the predicted values from the predictor variables (in our case NIR, e-tongue, e-nose and fusion data) per sample. It resulted in the different observation number per predictable attribute. In the case of the e-nose, the results of method four were used to build the PLSR models. The number of the latent variables (LV) were chosen after the model built using the aforementioned cross-validation method. The optimal number of latent variables was determined by the lowest value of root mean square error of prediction (RMSECV) separately for each tested attribute, making sure the LVs in the final model did not exceed the number of independent samples. All PCA, LDA, and PLSR models and data pre-treatments for NIR spectroscopy, e-tongue, and e-nose were utilized in the R-project software (v4.0.2) using the R-studio (v1.3.1093).

## 3. Results

### 3.1. Results of the Microscopic Pollen Analysis

From pollen analysis, three of the samples were 100% monofloral, while two samples contained pollen of other plant species besides the main pollen. The results of the microscopic pollen determination are summarized in Table 1.

### 3.2. Results of Bee Pollen the Colour Measurement

The lightness values (L*), the green-red values (a*) the blue-yellow values (b*), and chroma and hue angle of bee pollen samples are summarized in Table 2.

According to our results, the darkest sample was the one that originated mainly from spiny plumeless thistle with an L* value of 33.4. The lightest sample was rapeseed bee pollen with an L* value of 66.3. The a* values of the samples ranged from +0.9 to +15.6. The positive sign indicated that the shades of samples were closer to red than green. However, these values were relatively close to zero, so the green-red colour range could be characterized by low saturation. The b* values of the samples ranged from 17.1 to 64.5, so yellow dominated in the blue-yellow range in all cases. The b* of the sample mainly from spiny plumeless thistle was close to zero, but sunflower and rapeseed pollen had highly saturated colours. It can be assumed that the dominance of red and yellow was due to the presence of carotenoid and flavonoid compounds [50]. Based on the calculated value of chroma, the most vivid sample was sunflower pollen, followed by rapeseed, lakeshore bulrush, red clover, and spiny plumeless thistle. The hue angle values obtained for the samples ranged between 79.3 and 89.1, except for the sample mainly from spiny plumeless thistle.

The numerical values of the colour differences between the samples (ΔE*_ab_, ∆C*_ab_, ∆*h_ab_*) are summarized in Table 3. According to the spectral colour measurement, the ΔE*_ab_ for all sample pairs was above six, so there was a huge sensory difference between the samples [66]. Lakeshore bulrush and red clover pollen had similar saturation, so they showed a low ∆C*_ab_ value. The most vivid product was sunflower pollen, while the least vivid was the pollen mainly from spiny plumeless thistle; thus, these samples showed the highest ∆C*_ab_. In terms of quality, lakeshore bulrush and red clover show the smallest difference. The highest ∆*h_ab_* values were observed for spiny plumeless thistle, which had a significantly smaller yellow hue compared to the other samples.

### 3.3. Results of the Sensory Evaluation

The indicators of the sensory evaluation performance by software and the results of the quantitative descriptive profile analysis are presented based on our previous research [53]. After the 2-way ANOVA analysis, all sensory attributes were significantly different (*p* < 0.05), so there were no attributes that were not used for subsequent data analysis. The 2-way ANOVA model was the following: attribute = sample + assessor + sample × assessor. According to the Tucker-1 analysis and Manhattan plots, the panel showed a good performance and gave similar responses for the same sensory attribute. The panel members were located very close to each other on the outer ellipse, for except two attributes (after taste intensity and sour odour intensity). Assessors reached high explained variance using just the first two or three principal components; it can be deduced that panel members agreed well between the evaluated attributes. The MSE values show how similar are the given values of an assessor to the same stimulus, which means how consistent the assessor is. During our study, most of the MSE values were close to the zero, consequently having very good repeatability, so the repeatability of the panel was good. The MAM-CAP table presents the panel performance. The MAM-CAP table showed that the panel was generally well trained. All *F*-Prod and *F*-Disag values proved to be discriminant (*F*-Prod *p* < 0.05, *F*-Disag *p* > 0.05), and all attributes can be used in further analysis. With the exception of aftertaste and cohesiveness, the *F*-Scal value was adequate for all sensory characteristics (*F*-Scal *p* > 0.05). The root mean square error (RMSE) gives an indication of panel repeatability. With the exception of mouthcoating, all sensory attributes were very good (RMSE ≤ 3.45) (Table 4).

Five pollen samples were evaluated by the panel and were completely characterized by appearance, odour, taste/flavour, and texture parameters. Based on the results, there was a significant difference between the samples in 17 of the 18 sensory attributes. There was no difference between the samples in the aftertaste alone (Table 5).

### 3.4. Results of the NIR

**Figure 2:**
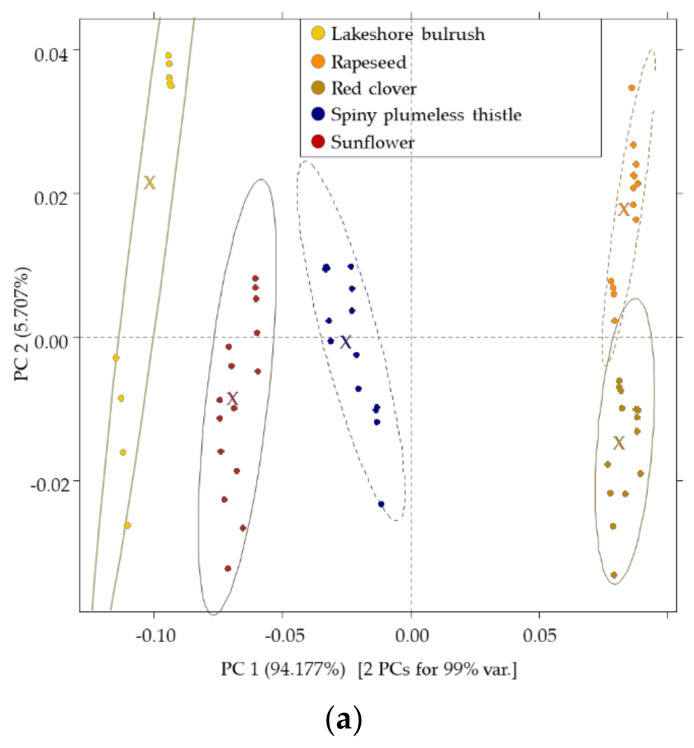

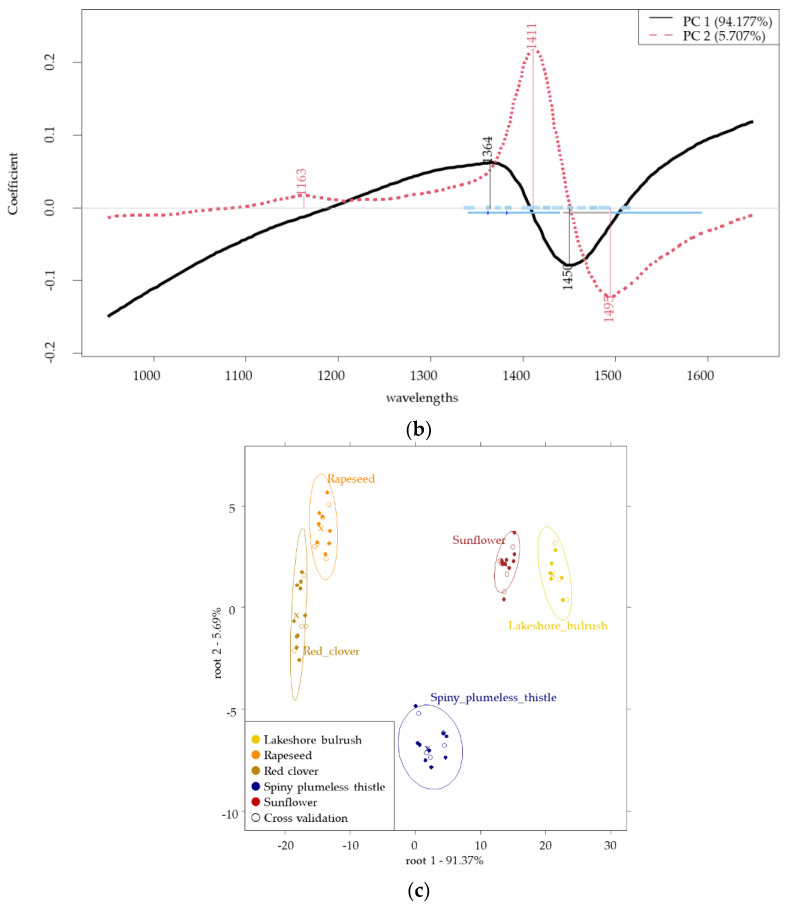
Results of the near-infrared spectroscopy: (**a**) PCA score plot NIRS (*n* = 65); (**b**) PCA loadings of the NIRS; (**c**) LDA score plots built for the classification of botanical groups with 95% confidence interval ellipses. x denotes the center of the ellipses, on figure (**c**) solid point are for the training set, and hollow points are for the validation set.

The score plot of the built PCA model showed a pattern of separation of samples from each other mainly through PC1, which described 94.177% of the total variance (Figure 2a). In the formation of PC1, the absorption bands 1364 nm and 1450 nm had the highest role (Figure 2b). While in the formation of PC2, the absorption bands of 1163 nm, 1415 nm, and 1495 nm had the highest role. LDA model showed correct classification for all the groups, resulting in 100% average recognition and prediction, which explains the distinct separation in the LDA score plot (Figure 2c).

Results of the regression models built on the properties of sensory profile analyses and colour parameters of pollen samples based on the data from NIR spectroscopy are shown in Table 6. The best prediction abilities were in the case of the homogeneity of the surface parameter, floral taste intensity, hay odour intensity, and L*. The R^2^Tr results of these parameters were in the range of 0.94–0.98, while R^2^CV in the range of 0.80–0.84. RPD results were above 2. Sweet odour intensity showed the lowest prediction accuracy using NIR data. In the case of the other parameters, R^2^Tr was higher than 0.59, but in some cases, the R^2^CV was worse.

### 3.5. Results of the Electronic Nose

**Figure 3:**
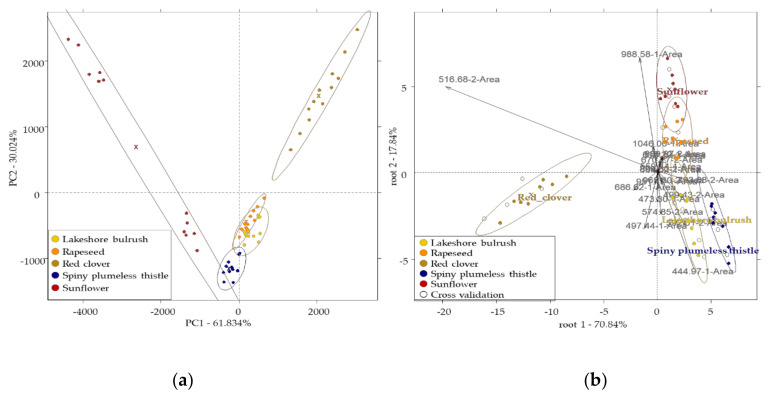
Results of the electronic nose using all the methods (*n* = 60): (**a**) PCA score plot; (**b**) LDA score plots built for the classification of botanical groups with 95% confidence interval ellipses. x denotes the center of the ellipses, on figure (**b**) solid point are for the training set, and hollow points are for the validation set.

PCA results of the electronic nose for using the chosen 20 most selective sensors showed that the method did not have an effect on the variance of the groups with the exception of the sunflower sample, where a scatter could be detected. This implies that the discrimination was more temperature-dependent than the sample volume because the points within the sunflower confidence ellipse showed that the points on the upper part are 40 °C and on the lower part are 60 °C (Figure 3a). PC 1 described 61.834% of the variance, where the separation pattern of the sunflower and red clover from each other and from the spiny plumeless thistle, rapeseed, and lakeshore bulrush groups could be observed. From the confidence ellipses in the PCA plot, Sunflower and Red clover samples showed the largest visual inter-group separation pattern. Some overlapping could, however, be seen between rapeseed and lakeshore bulrush samples and spiny plumeless samples. A similar separation pattern was also observed in the LDA plot, and the LDA model provided 100% accuracy for the classification of botanical groups when using results of all the methods (Figure 3b).

The separation of the sample groups can be observed on both root 1 (70.84%) and root 2 (17.84%), where 516.65-2A and 686.62-1A contributed mostly to root 1, and also to the discrimination of red clover samples. Sensor 516.65-2A can be assigned to the presence of Pent-2-ene, methyl chloride, methanol (alcoholic, pungent aroma), diethyl-ether (pungent, sweet aroma), and pentane compounds. Sensor 686.62-1A can be assigned to the compounds of 4-Difluorobenzene, 2-Methyl-1-propanethiol (onion, sulphurous, shallot, mustard, vegetable aroma), pent-1-en-3-ol (pungent, tropical, horseradish, green, vegetable, bitter, fruity aroma), pentan-2-on (alcohol, ethereal, fruit, sweet, woody aroma) and methyl isobutyrate. Sensor 793.68-2A highly contributed to the formation of root 1 and separation of rapeseed samples. Sensor 793.68-2A can be assigned to the presence of dimethyl disulphide (cabbage, onion, putrid, sulphurous aroma) and 1-Chloropentane (green plant aroma). Sensor 592.01-2A also contributed to root 1 and to the separation of the two polyfloral samples. This sensor can be associated with the compound of propane-2-on (fruity, glue, solvent aroma), and methyl acetate (blackcurrant, ethereal, fruity, solvent aroma). Through root 2, we can see mainly the separation of the two polyfloral samples from rapeseed and sunflower pollen samples. Sensors 998.58-1A contributed mostly to root 2 and to the separation of the sunflower and rapeseed samples. Sensor 1046.00-1A also contributed to the separation of these two pollen types. Sensor 998.58-1A associated with the compound of 3-menthene (earthy, herbaceous aroma), 4-Hydroxy-1-methylpiperidine (earthy, musty aroma), 3-Octanone (butter, herbaceous, resiny aroma), 6-Methyl-5-hepten-2-one (vinyl, woody, citrus, boiled fruit, and rubber) and phenol (phenolic, medicinal aroma). Sensor 1046.00-1A can be assigned to the presence of Dipropylene glycol, (s,p) (spicy aroma), DL-3-Aminoisobutyric acid, trimethylsilyl ester (medicinal aroma), (Z)-2-octenal (earthy, green, leafy, aroma), butanoic acid, 3-methylbutyl ester (fruity aroma), and ethyl furoate (floral, plum aroma). Sensor 444.97-1A, and 516.65-2A also contributed root 2, and the separation of red clover and polyfloral pollen samples, respectively.

Analysing the data separately for the four different methods showed that the data acquired by method four resulted in the best classification. In this case, all the samples were classified correctly.

Partial least-squares regression results of the sensory attributes and colour parameters using the electronic nose data of method 4 provided the best result in the case of the sweet taste intensity, mouthcoating, a* and ∆C*_ab__._ Determination coefficient of the training was in the range of 0.97–0.79, while for the validation it was in the range of 0.62–0.89. The RPD results were the best in the case of sweet taste intensity. In general, the results of the PLSR obtained using the e-nose were worse than those obtained using NIR. Sour taste intensity could not be predicted using the e-nose (Table 7).

### 3.6. Results of the Electronic Tongue

**Figure 4:**
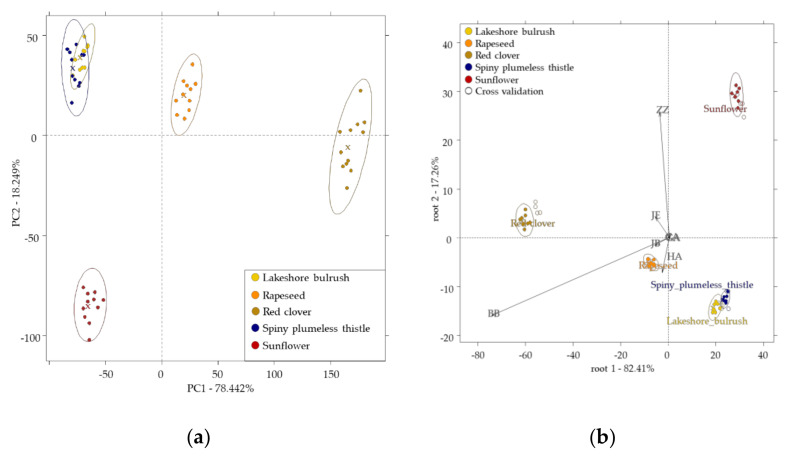
Results of the electronic tongue using all the methods (*n* = 55): (**a**) PCA score plot; (**b**) LDA score plot built for the classification of botanical groups with 95% confidence interval ellipses after additive correction relative to all samples. x denotes the center of the ellipses, on figure (**b**) solid point are for the training set, and hollow points are for the validation set.

Results of the PCA using electronic tongue data showed a distinct separation tendency of the pollen groups. Samples from sunflower, red clover, and rapeseed were completely separated from the other groups, while samples from lakeshore bulrush and spiny plumeless thistle overlapped with each other but were separated from the other three groups (Figure 4a). This pattern was also observed in the LDA plot, where 82.41% of the variance was expressed in the root1 (Figure 4b).

This phenomenon was also proven by the LDA results. The average recognition and prediction abilities were 99.01% and 94.36%, respectively. The classification of the groups showed that samples from sunflower, red clover, and rapeseed were classified correctly. Lakeshore bulrush pollen was misclassified belonging to the pollen sample mainly from spiny plumeless thistle with misclassification rates of 4.95% and 9.91% during training and cross-validation, respectively, while spiny plumeless thistle pollen was classified correctly during training, but showed misclassification belonging to lakeshore bulrush in cross-validation with 18.26% misclassification rate.

Partial least square regression results regressing on the sensory attributes and colour parameters using the electronic nose provided the best result in the case of hay taste intensity, mouthcoating, global odour intensity, floral taste intensity, and sweet taste intensity. The R^2^Tr values ranged between 0.98 and 0.88, while R^2^CV results ranged from 0.77 to 0.97 for these parameters. These results were better than the results of the electronic nose, and similar to results obtained by NIR. Electronic tongue results of PLSR showed the worst model for the colour hue parameter with 0.09 R^2^CV (Table 8).

### 3.7. Partial least Square Regression Results of the Fusion of NIR Spectroscopy, e-Nose, e-Tongue Methods to Regress on the Sensory Attributes and Colour Parameters

Partial least-squares regression results of the sensory and colour attributes using the fused data of NIR spectroscopy, e-nose and e-tongue showed better accuracies compared to the independent models built for instruments. R^2^Tr results were in the range of 0.80–0.99, and R^2^CV results in the range of 0.55–0.99. Compared to the independent models built for the instruments, it was also observed that there was a generally decreasing RMSEC but with increasing RPD values. The worst model was the one obtained for aftertaste intensity but all the other parameters were predicted with high determination coefficient. The best model was obtained for mouthcoating, which was also one of the best models in the case of e-nose and e-tongue (Table 9).

## 4. Discussion

Our results support the findings of other researchers [67,68], according to which pollen loads of different plant species cannot be sorted perfectly on the basis of colour, whereas pollen of certain species have the same colour and the colour of a pollen load can change due to oxidation processes. It is important to characterize the colour of pollen loads and quantify the differences between samples. The tristimulus CIE L*a*b* colour measurement system based on additive colour mixing is widely used for this purpose. Test samples can be characterized by colour coordinates (chroma and hue) calculated from L*, a*, b* values. Castiglioni et al. Reference [26] determined L*, a* and b* values of bee pollen from 17 different plant species and found that the coordinates of the samples were between the following ranges: L* = 28.6–67.6; a* = −1.9–22.3; b* = 12.0–69.4. Our results are within these ranges in all cases. In order to further fully characterize the pollen samples, it is advisable to provide data on the differences of distances between colour coordinates (ΔE*_ab_, ∆C*_ab_, ∆*h_ab_*). From the spatial location and relative distance of colour points determined by measuring of the samples, we can draw conclusions on the direction and magnitude of colour differences between the samples, as well as on the sensometric perceptible colour difference [37].

Based on the sensory results, the sensory panel was well trained and was characterized by good discrimination, good repetition, and good agreement, which were verified by performance indicators (PanelCheck, MAM-CAP table). Monitoring the performance evaluation of the sensory panel provides the basis for reliable results and comparison with instrumental results.

Results of the NIR measurement showed similar spectra of the liquified samples; however, the samples could be separated using LDA. The visual similarity among spectra can be explained by the high absorbance of water in the NIR region. The bands contributed to PC1 as per 1364 nm can be assigned to the non- or less-H-bonded water with the domination of free -OH vibrations, while those of 1450 nm can be assigned to the first overtone O-H stretches [69]. Wavelength ranges around 1170 nm correspond to the second-overtone region C-H stretching bands [70]. Previous studies showed that wavelengths close to 1412 are related to less hydrogen-bonded water, while wavelength bonds around 1490 nm are assigned to water containing more H bonds [69]. PLRS results of NIR provided good results in the case of the L* parameter (R^2^Tr = 0.94, R^2^CV = 0.8); similar results were found from Polish researchers using Raman spectroscopy [71]. The homogeneity of the surface also showed a high determination coefficient predicted using NIR data; this could be due to the fact that high differences were found also within the sensory attribute itself.

E-nose results showed that the temperature of the sample was more influencing than the volume (3 mL/5 mL) of the sample used for analysis. This effect was observed in the case of the sunflower sample in particular. The reason for the higher effect of temperature could be because the volatile compounds are more intensely expressed at higher temperatures (60 °C) than at lower temperatures (40 °C). Sunflower samples were particularly susceptible to this influence, probably because it contains volatiles that are more sensitive to temperature. E-nose provided similar classification results to NIR (100% correct classification of the sample groups) and better classification results than e-tongue (94.36% correct classification in validation for the five sample groups) for the botanical origin identification. Our results showed that the misclassification in the case of the e-tongue was found between samples containing the same bee pollen specie (*Carduus acanthoides*). This suggests that the sensors of the electronic tongue may be more sensitive to this specie then e-nose and NIR. PLRS results were worse than the other two methods. The best results were obtained for the prediction of sweet taste. From e-nose and the sensory results, compounds associated with sweet aroma were also found to be significantly different among four of the samples by the electronic nose and the sensory results.

The e-tongue showed the discrimination of the unifloral pollen samples, but misclassification was found between the two polyfloral pollen samples. This misclassification may have originated from the fact that both polyfloral pollens contained *Carduus acanthoides* pollen, and e-tongue sensors are more sensitive to this specie, and in the liquid its presence is more expressed than in the head-space of the e-nose. The results of the PLSR regression model provided one of the best results in the case of the mouthcoating (R^2^CV = 0.97), floral taste intensity (R^2^CV = 0.85), and good results were found for sweet taste (R^2^CV = 0.77). These results can be explained by the sweet (diethyl-ether, pentan-2-on) and floral aroma (ethyl furoate) property-related chemical compounds identified by the e-nose coupled GC-MS.

The fusion of the three methods, NIR, e-nose, and e-tongue, provided improved results compared to the results of the independent method. Similar to the e-nose and e-tongue, the best results were obtained for the mouthcoating (R^2^CV = 0.99), floral taste intensity (R^2^CV = 0.97), sweet taste intensity (R^2^CV = 0.96), global odour and global taste intensity (R^2^CV = 0.96). This can be assigned to the rich aroma profile of the samples proven by the identified aroma compounds by the e-nose. Volatile compounds have been also found in bee pollen by other researchers, proving the rich aroma profile of the product [72,73,74]. The worst prediction model was obtained for the aftertaste (R^2^CV = 0.55), which can be explained on one hand by the fact that no significant difference was found in the case of the aftertaste intensity during the sensory profile analysis. On the other hand, the aftertaste perception begins after swallowing [75], which cannot be modelled by the e-senses and NIR.

The emphasis of this work was the demonstration of these methods, so a limited set of samples was used. A larger number of samples is recommended for similar studies in the future.

## 5. Conclusions

In our work, the multidimensional pattern of bee pollen was analysed by chemometric methods with a sensometric fusion approach. Our results showed that, in addition to the microscopic method, sensory, NIR spectroscopy, the electronic nose, and electronic tongue can also be used to distinguish uterine pollen samples with the present test samples and under the test conditions presented. To generalize the results, it is recommended to eliminate limitations using a large data set, independent, and highly variable bee pollen samples. In order to be able to obtain more robust and reliable results, there could be a demand for a database containing results of electronic sensory and NIR data. This database should be composed of bee pollens from different botanical origin. Moreover, building up such a database could be used for independent validation of our results. GC–MS coupled with the e-nose can provide comprehensive aroma analysis for bee pollen, which also allows the analysis of the main aroma components. Our result suggests that in the future, these could be promising tools for the prediction of the geographical origin of the bee pollen samples.

## Figures and Tables

**Table 1:** Results of the microscopic pollen determination.

Sample	Main Plant Species		Proportion of Main Plant Species	Minor Plant Species
	Common Name	Scientific Name		
1	Lakeshore bulrush	*Schoenoplectus lacustris* (L.) *Palla*	80%	*Cornus sanguinea*
				*Plantago lanceolata*
				*Zea mays*
				*Robinia pseudoacacia*
				*Carduus acanthoides*
				*Convolvulus arvensis*
2	Sunflower	*Helianthus annuus*	100%	−
3	Red clover	*Trifolium pretense*	100%	−
4	Rapeseed	*Brassica napus*	100%	−
5	Spiny plumeless thistle	*Carduus acanthoides* L.	85%	*Trifolium pratense*

**Table 2:** The L*, a*, b*, C*_ab_ and *h_ab_* values of bee pollen samples.

Samples	L*	a*	b*	C*_ab_	*h_ab_*
Lakeshore bulrush	59.6 ± 0.4 ^b^	6.0 ± 0.1 ^c^	44.2 ± 0.9 ^c^	44.6	82.3
Sunflower	58.1 ± 0.2 ^c^	12.2 ± 0.1 ^b^	64.5 ± 0.2 ^a^	65.6	79.3
Red clover	50.7 ± 0.4 ^d^	5.8 ± 0.1 ^c^	40.7 ± 0.4 ^d^	41.1	81.9
Rapeseed	66.3 ± 0.5 ^a^	0.9 ± 0.2 ^d^	54.6 ± 0.4 ^b^	54.6	89.1
Spiny plumeless thistle	33.4 ± 0.3 ^e^	15.6 ± 0.2 ^a^	17.1 ± 0.3 ^e^	23.1	47.6

^a–e^ indication of homogeneous and heterogeneous groups tested by Tukey-HSD test at 95% confidence level.

**Table 3:** The ΔE*_ab_, ∆C*_ab_ and ∆*h_ab_* values of bee pollen samples.

	Lakeshore Bulrush	Sunflower	Red Clover	Rapeseed	Spiny Plumeless Thistle
Lakeshore bulrush	–	21.28 *	9.57 *	13.38 *	38.90 *
		21.03 **	3.50 **	10.00 **	21.46 **
		3.0 ***	0.4 ***	6.8 ***	34.7 ***
Sunflower		–	25.73 *	17.12 *	53.56 *
			24.53 **	11.03 **	42.49 **
			2.6 ***	9.8 ***	31.7 ***
Red clover			–	21.46 *	30.86 *
				13.50 **	17.96 **
				7.2 ***	34.3 ***
Rapeseed				–	52.01 *
					41.5 ***
					31.46 **
Spiny plumeless thistle					–

*: ΔE*_ab_; where, ΔE*_ab_ < 1.5: not perceptible; 1.5< ΔE*_ab_ < 3.0: perceptible; 3.0 < ΔE*_ab_ < 6.0: well perceptible; 6.0 < ΔE*_ab_: huge; **: ∆C*_ab_; ***: ∆*h_ab_*.

**Table 4:** The sensory panel performance MAM-CAP table. The first column contains the mean of the panel for each attribute. The four following columns are respectively: F statistics of discrimination (*F*-Prod), scaling heterogeneity (*F*-Scal), disagreement (*F*-Disag), and repeatability (root mean squares of error, RMSE).

Attribute	*F*-Prod	*F*-Scal	*F*-Disag	RMSE
brightness	1360.05	0.42	1.55	2.33
colour hue	7303.81	1.70	0.84	2.21
homogeniety of surface	275.96	1.25	1.06	3.27
global odour intensity	252.87	0.66	1.50	2.89
sweet odour intensity	279.68	0.39	1.38	2.58
sour odour intensity	116.52	1.70	1.35	1.56
floral odour intensity	46.71	1.07	1.24	1.95
hay odour intensity	208.2	1.00	1.50	2.15
global taste intensity	44.47	1.24	1.48	3.12
sweet taste intensity	120.03	1.11	1.42	3.35
sour taste intensity	844.91	1.20	1.37	2.33
floral taste intensity	8.83	1.10	1.66	2.37
hay taste intensity	162.88	1.60	1.15	1.85
off-taste intensity	344.46	0.65	1.29	3.45
aftertaste intensity	4.68	106.95	0.32	0.64
hardness	81.91	1.61	1.01	2.21
cohesiveness	3572.56	7.19	1.55	2.08
mouthcoating	579.97	1.10	1.37	66.86

**Table 5:** Sensory attributes of bee pollen (quantitative descriptive analysis, QDA).

Attributes	Lakeshore Bulrush (Mean ± Std.)	Sunflower (Mean ± Std.)	Red Clover (Mean ± Std.)	Rapeseed (Mean ± Std.)	Spiny Plumeless Thistle (Mean ± Std.)
brightness	45.5 ± 3.9 ^c^	29.6 ± 3.7 ^d^	68.9 ± 2.1 ^b^	11.5 ± 2.0 ^e^	84.3 ± 3.5 ^a^
colour hue	40.9 ± 2.7 ^c^	23.9 ± 2.4 ^d^	61.7 ± 2.0 ^b^	11.4 ± 2.0 ^e^	94.1 ± 1.9 ^a^
homogeniety of surface	62.9 ±3.0 ^c^	64.5 ± 5.1 ^c^	80.8 ± 1.7 ^b^	85.6 ± 3.5 ^a^	81.3 ± 1.9 ^b^
global odour intensity	85.6 ± 3.4 ^b^	63.8 ± 2.9 ^d^	70.0 ± 3.4 ^c^	88.0 ± 3.2 ^a^	86.5 ± 3.4 ^a,b^
sweet odour intensity	63.0 ± 3.0 ^d^	67.0 ± 2.8 ^c^	56.7 ± 2.7 ^e^	71.3 ± 2.6 ^b^	82.4 ± 2.2 ^a^
sour odour intensity	17.3 ± 3.2 ^a^	5.5 ± 1.7 ^c^	6.0 ± 2.1 ^c^	10.4 ± 1.1 ^b^	7.1 ± 2.5 ^c^
floral odour intensity	11.8 ± 2.4 ^a^	6.3 ± 2.3 ^c^	3.2 ± 2.8 ^d^	8.7 ± 1.8 ^b^	2.8 ± 2.7 ^d^
hay odour intensity	20.3 ± 3.7 ^a^	11.7 ± 2.9 ^c^	2.8 ± 2.5 ^d^	14.8 ± 2.5 ^b^	2.4 ± 2.0 ^d^
global taste intensity	87.8 ± 3.9 ^b^	92.0 ± 2.8 ^a^	83.3 ± 4.1 ^c^	81.8 ± 3.8 ^c,d^	79.8 ± 2.8 ^d^
sweet taste intensity	86.2 ± 4.8 ^a^	64.4 ± 3.5 ^d^	72.8 ± 5.0 ^b^	68.4 ± 2.8 ^c^	84.1 ± 3.9 ^a^
sour taste intensity	7.4 ± 3.5 ^e^	14.4 ± 3.6 ^d^	47.7 ± 4.1 ^c^	69.1 ± 2.4 ^a^	63.4 ± 5.5 ^b^
floral taste intensity	5.5 ± 2.8 ^b^	5.5 ± 3.0 ^b^	8.3 ± 2.3 ^a^	9.0 ± 1.8 ^a^	8.3 ± 3.3 ^a^
hay taste intensity	4.4 ± 1.6 ^c^	15.5 ± 3.0 ^a^	7.6 ± 2.5 ^b^	4.0 ± 2.6 ^c^	4.8 ± 0.6 ^c^
off-taste intensity	42.3 ± 5.5 ^b^	64.6 ± 5.6 ^a^	32.8 ± 4.6 ^c^	32.5 ± 5.8 ^c^	0.0 ± 0.0 ^d^
aftertaste intensity	0.1 ± 0.4 ^a^	0.0 ± 0.0 ^a^	0.0 ± 0.4 ^a^	0.0 ± 0.0 ^a^	0.4 ± 1.3 ^a^
hardness	10.5 ± 1.0 ^c^	8.4 ± 2.3 ^d^	11.0 ± 1.8 ^c^	13.4 ± 2.0 ^b^	18.4 ± 3.6 ^a^
cohesiveness	10.4 ± 0.8 ^b,c^	8.9 ± 3.3 ^c^	12.1 ± 2.3 ^b^	5.9 ± 2.0 ^d^	74.6 ± 5.9 ^a^
mouthcoating	76.8 ± 3.4 ^b^	86.8 ± 3.6 ^a^	35.9 ± 4.8 ^e^	63.8 ± 2.5 ^d^	71.0 ± 4.1 ^c^

^a–e^ indication of homogeneous and heterogeneous groups tested by Tukey-HSD test at 95% confidence level.

**Table 6:** Results of the regression models built on the properties of sensory profile analyses and colour parameters of pollen samples based on data of the NIR spectroscopy at the wavelength range of 950–1650 after Savitzky-Golay smoothing and MSC pretreatment.

Predicted Variable	R^2^Tr	RMSEC	R^2^CV	RMSECV	RPD	Number of Latent Variables	Number of Observations
brightness	0.81	11.53	0.54	17.75	1.49	3	58
colour hue	0.85	11.14	0.7	15.87	1.84	3	58
homogeneity of surface	0.98	1.26	0.85	3.53	2.62	5	57
global odour intensity	0.9	3.15	0.49	7.08	1.41	5	59
sweet odour intensity	0.06	8.47	−0.25	9.75	0.90	1	63
sour odour intensity	0.59	2.49	0.14	3.61	1.09	3	57
floral odour intensity	0.95	0.7	0.75	1.62	2.03	5	55
hay odour intensity	0.96	1.28	0.84	2.75	2.48	5	56
global taste intensity	0.95	0.95	0.75	2.19	2.03	5	59
sweet taste intensity	0.61	5.11	0.2	7.33	1.13	3	61
sour taste intensity	0.96	5.11	0.67	14.2	1.75	5	55
floral taste intensity	0.96	0.3	0.84	0.62	2.5	4	61
hay taste intensity	0.77	2.08	0.2	3.91	1.13	4	61
off-taste intensity	0.95	4.83	0.74	10.64	2.00	5	58
aftertaste intensity	0.84	0.05	0.7	0.07	1.84	3	61
hardness	0.95	0.81	0.69	1.97	1.8	5	59
cohesiveness	0.95	6.17	0.68	15.39	1.79	5	58
mouthcoating	0.66	10.34	0.52	12.36	1.45	1	65
L*	0.94	2.76	0.80	4.9	2.27	4	57
a*	0.85	1.78	0.50	3.57	1.42	4	55
b*	0.94	4.28	0.65	9.94	1.71	5	59
∆C*_ab_	0.78	6.92	0.59	9.43	1.58	3	61
∆*h_ab_*	0.93	3.68	0.61	8.96	1.61	5	58

**Table 7:** Results of the regression models built on the properties of sensory profile analyses and colour parameters of pollen samples based on data of the electronic nose.

Predicted Variable	R^2^Tr	RMSEC	R^2^CV	RMSECV	RPD	Number of Latent Variables	Number of Observations
brightness	0.66	15.38	0.44	19.44	1.39	2	15
colour hue	0.65	17.42	0.42	22.12	1.36	2	15
homogeneity of surface	0.69	5.23	0.00	9.22	1.03	2	15
global odour intensity	0.74	5.02	0.62	6.25	1.69	2	15
sweet odour intensity	0.68	4.86	0.53	5.85	1.52	2	15
sour odour intensity	0.56	2.88	0.37	3.46	1.31	2	15
floral odour intensity	0.72	1.79	0.27	2.84	1.21	2	15
hay odour intensity	0.87	2.46	0.26	5.52	1.21	2	15
global taste intensity	0.49	3.17	0.27	3.76	1.21	2	15
sweet taste intensity	0.92	2.39	0.89	2.81	3.16	2	15
sour taste intensity	0.48	18.2	−0.06	26.65	1.00	2	15
floral taste intensity	0.58	1.00	0.06	1.52	1.07	2	15
hay taste intensity	0.78	2.02	0.69	2.39	1.86	2	15
off-taste intensity	0.62	12.87	0.38	16.55	1.31	2	15
aftertaste intensity	0.67	0.08	0.49	0.09	1.45	2	15
hardness	0.54	2.31	0.32	2.81	1.25	2	15
cohesiveness	0.61	16.33	0.41	20.12	1.34	2	15
mouthcoating	0.97	2.91	0.87	5.66	2.88	2	15
L*	0.68	6.38	0.5	8.25	1.46	2	15
a*	0.81	2.26	0.62	3.15	1.69	2	15
b*	0.76	7.84	0.61	9.92	1.65	2	15
∆C*_ab_	0.79	6.45	0.67	8.14	1.79	2	15
∆*h_ab_*	0.62	9.04	0.41	11.14	1.35	2	15

**Table 8:** Results of the regression models built on the properties of sensory profile analyses and colour parameters of pollen samples based on data of the electronic tongue.

Predicted Variable	R^2^Tr	RMSEC	R^2^CV	RMSECV	RPD	Number of Latent Variables	Number of Observations
brightness	0.68	14.91	0.22	22.88	1.14	5	46
colour hue	0.44	21.84	0.09	27.56	1.06	3	43
homogeneity of surface	0.73	4.87	0.54	6.26	1.49	4	50
global odour intensity	0.95	2.31	0.91	3.02	3.32	4	47
sweet odour intensity	0.76	4.39	0.58	5.83	1.55	4	45
sour odour intensity	0.78	1.92	0.57	2.67	1.54	5	49
floral odour intensity	0.66	1.97	0.16	3.05	1.11	5	47
hay odour intensity	0.54	4.75	0.16	6.37	1.11	4	45
global taste intensity	0.85	1.71	0.61	2.69	1.62	5	51
sweet taste intensity	0.88	2.96	0.77	4.11	2.10	4	44
sour taste intensity	0.65	14.47	0.43	18.4	1.34	4	51
floral taste intensity	0.90	0.46	0.85	0.57	2.61	4	44
hay taste intensity	0.98	0.55	0.97	0.69	5.94	4	44
off-taste intensity	0.88	6.96	0.68	10.88	1.80	5	49
aftertaste intensity	0.50	0.09	0.04	0.12	1.03	3	44
hardness	0.83	1.34	0.66	1.87	1.74	5	49
cohesiveness	0.59	16.76	0.21	22.94	1.14	4	45
mouthcoating	0.98	2.16	0.97	3.20	5.42	4	44
L*	0.62	6.46	0.00	10.27	1.01	5	46
a*	0.72	2.67	0.56	3.32	1.53	3	41
b*	0.77	7.57	0.44	11.73	1.35	5	46
∆C*_ab_	0.72	7.64	0.46	10.47	1.37	3	45
∆*h_ab_*	0.67	7.62	0.14	12.00	1.09	5	46

**Table 9:** Results of the regression models built on the properties of sensory profile analyses and colour parameters of pollen samples based on data of the fusion of NIR, EN, and ET.

Predicted Variable	R^2^Tr	RMSEC	R^2^CV	RMSECV	RPD	Number of Latent Variables	Number of Observations
brightness	0.99	2.14	0.96	4.61	5.06	5	70
colour hue	0.97	4.80	0.90	9.08	3.20	5	70
homogeniety of surface	0.99	0.77	0.98	1.40	6.67	5	70
global odour intensity	0.99	1.09	0.96	2.05	4.82	5	70
sweet odour intensity	0.98	1.21	0.94	2.13	3.99	5	70
sour odour intensity	0.98	0.66	0.94	1.07	4.09	5	70
floral odour intensity	0.97	0.58	0.91	1.02	3.34	5	70
hay odour intensity	0.97	1.15	0.91	2.03	3.42	5	70
global taste intensity	0.99	0.46	0.96	0.90	4.95	5	70
sweet taste intensity	0.99	0.87	0.96	1.69	5.12	5	70
sour taste intensity	0.99	2.45	0.97	4.41	5.65	5	70
floral taste intensity	0.99	0.14	0.97	0.24	6.25	4	70
hay taste intensity	0.99	0.47	0.96	0.88	4.92	5	70
off-taste intensity	0.98	2.96	0.93	5.62	3.73	5	70
aftertaste intensity	0.80	0.06	0.55	0.09	1.49	3	70
hardness	0.98	0.51	0.92	0.94	3.61	5	70
cohesiveness	0.97	4.87	0.89	8.72	2.98	5	70
mouthcoating	0.99	0.81	0.99	1.43	12.10	5	70
L*	0.96	2.14	0.88	3.85	2.89	5	70
a*	0.97	0.91	0.90	1.57	3.27	5	70
b*	0.98	2.41	0.91	4.63	3.44	5	70
∆C*_ab_	0.98	1.99	0.92	3.91	3.64	5	70
∆*h_ab_*	0.96	2.77	0.88	4.92	2.93	5	70
